# Livestock keeping, mosquitoes and community viewpoints: a mixed methods assessment of relationships between livestock management, malaria vector biting risk and community perspectives in rural Tanzania

**DOI:** 10.1186/s12936-024-05039-1

**Published:** 2024-07-17

**Authors:** Yohana A. Mwalugelo, Winifrida P. Mponzi, Letus L. Muyaga, Herieth H. Mahenge, Godfrey C. Katusi, Faith Muhonja, Dickens Omondi, Alfred O. Ochieng, Emmanuel W. Kaindoa, Fred A. Amimo

**Affiliations:** 1 Environmental Health and Ecological Sciences Department, Ifakara Health Institute, P. O. Box 53, Ifakara, Tanzania; 2 Department of Biomedical Sciences, Jaramogi Oginga Odinga University of Science and Technology, P. O. Box 210, Bondo, 40601 Kenya; 3 School of Biodiversity, Animal Health and Comparative Medicine, University of Glasgow, Glasgow, G12 8QQ UK; 4 The Nelson Mandela, African Institution of Science and Technology, School of Life Sciences and BioEngineering, Tengeru, Arusha, United Republic of Tanzania; 5 School of Public Health, Amref International University, P.O. Box 27691-00506, Nairobi, Kenya; 6 Department of Biological Sciences, Jaramogi Oginga Odinga University of Science and Technology, P.O. Box 210, Bondo, 40601 Kenya; 7 Wits Research Institute for Malaria, School of Pathology, Faculty of Health Sciences, University of the Witwatersrand and the Centre for Emerging Zoonotic and Parasitic Diseases, National Institute for Communicable Diseases, Johannesburg, South Africa

**Keywords:** Livestock management, Zooprophylaxis, Zoopotentiation, Mosquito biting risk, Blood meal, Malaria transmission, Community perspective

## Abstract

**Background:**

Livestock keeping is one of the potential factors related to malaria transmission. To date, the impact of livestock keeping on malaria transmission remains inconclusive, as some studies suggest a zooprophylactic effect while others indicate a zoopotentiation effect. This study assessed the impact of livestock management on malaria transmission risks in rural Tanzania. Additionally, the study explored the knowledge and perceptions of residents about the relationships between livestock keeping and malaria transmission risks in a selected village.

**Methods:**

In a longitudinal entomological study in Minepa village, South Eastern Tanzania, 40 households were randomly selected (20 with livestock, 20 without). Weekly mosquito collection was performed from January to April 2023. Indoor and outdoor collections used CDC-Light traps, Prokopack aspirators, human-baited double-net traps, and resting buckets. A subsample of mosquitoes was analysed using PCR and ELISA for mosquito species identification and blood meal detection. Livestock's impact on mosquito density was assessed using negative binomial GLMMs. Additionally, in-depth interviews explored community knowledge and perceptions of the relationship between livestock keeping and malaria transmission risks.

**Results:**

A total of 48,677 female *Anopheles* mosquitoes were collected. Out of these, 89% were *Anopheles gambiae *sensu lato (*s.l*.) while other species were *Anopheles funestus s.l*., *Anopheles pharoensis*, *Anopheles coustani,* and *Anopheles squamosus*. The findings revealed a statistically significant increase in the overall number of *An. gambiae s.l.* outdoors (RR = 1.181, 95%CI 1.050–1.862, *p* = 0.043). Also, there was an increase of the mean number of *An. funestus s.l.* mosquitoes collected in households with livestock indoors (RR = 2.866, 95%CI: 1.471–5.582, *p* = 0.002) and outdoors (RR = 1.579,95%CI 1.080–2.865, *p* = 0.023). The human blood index of *Anopheles arabiensis* mosquitoes from houses with livestock was less than those without livestock (OR = 0.149, 95%CI 0.110–0.178, *p* < 0.001). The majority of participants in the in-depth interviews reported a perceived high density of mosquitoes in houses with livestock compared to houses without livestock.

**Conclusion:**

Despite the potential for zooprophylaxis, this study indicates a higher malaria transmission risk in livestock-keeping communities. It is crucial to prioritize and implement targeted interventions to control vector populations within these communities. Furthermore, it is important to enhance community education and awareness regarding covariates such as livestock that influence malaria transmission.

## Background

In recent years, vector control tools, such as insecticide-treated nets (ITNs) and indoor residual spraying (IRS) have significantly contributed to the reduction of malaria transmission worldwide [1–4]. However, between 2015 and 2021 malaria control has stalled due to emergence of insecticide resistance, mosquitoes’ behavioural avoidance of insecticides and the inherent behavioural plasticity of some malaria vector species amongst malaria vectors that keeps them out of contact with indoor-based mosquito control methods [5–8]. Additionally, although *Anopheles stephensi* is originally an Asian vector, its emergence and spread in African regions, particularly in urban areas, indeed raise significant concerns due to its highly zoophilic behaviour [9–11].

Mosquito vectors are known to obtain blood from human and non-human hosts, such as livestock [12, 13]. Female mosquitoes identify their hosts by detecting host odours in the environment and then following these cues upwind from long or short ranges [14]. Blood meal is necessary for mosquitoes to develop their eggs [14]. Although there are varying blood feeding behaviours among mosquito species, some *Anopheles* species prefer to feed on humans, such as *Anopheles funestus *sensu stricto (*s.s*.), while other species prefer to feed on both animals and human beings including *Anopheles arabiensis* [12, 15].

Usually, the use of ITNs and IRS targets mosquitoes vectors that bite humans (anthropophilic) and that feed and rest indoor (endophagic and endophilic) [16, 17], but some malaria vectors exhibit different feeding and resting behaviours for there are some mosquitoes that bite at dusk and dawn, rest and feed outdoors (exophilic and exophagic respectively) and obtain alternative blood meal from other vertebrates [18, 19]. Exclusive human blood indices (proportion of mosquito samples which are positive for human blood) are extremely unusual in the majority of malaria vectors, indicating that some malaria vectors obtain blood meal from other vertebrates, most commonly cattle [12, 13, 20]. This is because cattle are often more easily accessible such that the host preferences of many female mosquito species show a high degree of plasticity, primarily as a result of environmental factors, such when preferred host species vanish or become inaccessible [12]. In some settings, non-human feeding and outdoor resting behaviours of *Anopheles* mosquitoes are common [12, 18]. This is mediated by various factors, such as presence of other animals nearby human dwellings, effective application of mosquito control interventions, such as ITNs to protect humans against mosquito bites, and the zoophilic behaviour of mosquito species especially *An. arabiensis* [18, 21]. The mosquito species which feed partially on human and livestock blood, enhance mosquitoes’ fitness, thus contributing to the persistence of malaria transmissions [22, 23].

Tanzania is one of the countries with the largest number of livestock in sub-Saharan Africa, comprising approximately 33.8 million cattle (98% of which are indigenous breeds), 24.5 million goats, 8.5 million sheep, 3.2 million pigs, and 87.7 million chickens [24]. Livestock keeping is one of the economic activities that plays an important role in poverty alleviation, food security enhancement, employment creation, and environmental conservation, particularly, in village settings [25]. Since livestock are not infected with *Plasmodium* parasites, for a long period of time, it has been proposed to establish interventions to control malaria by using the available livestock in societies by diverting malaria vector biting from humans, an intervention known as zooprophylaxis [26, 27]. In the process of assessing the effectiveness of livestock-based malaria interventions, there are still contradictions in the number of studies that have been conducted in different parts of the world. Some studies support a zooprophylaxis approach, while others go against it.

Livestock husbandry is one of the most important economic activities in rural areas of sub-Saharan Africa [25]. However, some evidence suggests that domestic livestock production at the household level may increase the risk of malaria transmission [28, 29]. It has been hypothesized that the presence of livestock, particularly cattle, provides alternative sources of blood meal for mosquitoes, thereby increasing mosquito density and their survival rate [26]. Some evidence shows that cattle contribute to higher mosquito density and malaria transmission [30, 31]. Furthermore, cattle keeping is responsible for the creation of suitable breeding habitats for malaria vectors through the creation of drinking water sites and hoof prints, especially during rainy seasons, resulting in a high mosquito population in communities with cattle [32, 33]. Another study in Burkina Faso [34] observed a positive correlation between donkeys and *Anopheles gambiae* abundance inside houses. This correlation raises an important question about the potential role of donkeys in attracting mosquitoes into human dwellings. These relationships could be utilized to develop effective strategies for malaria control.

Other studies, however, have provided contradictory results, indicating that keeping livestock may reduce the risk of malaria transmission. Mayagaya et al*.* [35] found that presence of cattle at household level significantly altered the *An. arabiensis* and *An. funestus *sensu lato (*s.l*.) species composition, feeding and resting behaviours of mosquitoes, thereby reducing the risks of malaria transmission in rural Tanzania. In that study, mosquitoes collected from households with cattle had lower human blood index and sporozoite rate than those without cattle. Furthermore, additional studies have also found that keeping cattle can divert mosquitoes that would otherwise feed on humans and the increased blood feeding on cattle reduces the human-mosquito contacts, hence reducing risk of malaria transmission [32, 36].

Overall, studies have shown contrasting perspectives on the relationship between livestock keeping and malaria transmission risks. Whereas studies suggest that livestock keeping poses risk to malaria transmission (zoopotentiation effect), others suggest that keeping livestock acts as a protective factor against malaria transmission (zooprophylaxis). In northern Tanzania, the use of insecticide-treated animals have been reported to have a significant effect on killing 50% of *An. arabiensis* mosquitoes up to 21 days for grazing cattle and 29 days for non-grazing cattle [21]. This information is vital when designing effective livestock-based control interventions against particular malaria vectors which may also be used alongside other core interventions such as the use of ITNs and IRS [36, 37]. It is clear that the impact of livestock on malaria transmission, distribution, and densities of potential malaria vectors is a complex issue that needs more investigation in different settings. These two contrasting findings formed a basis for this study to assess the impact of livestock management and keeping practices on malaria transmission risks in a selected village in South-eastern Tanzania.

Moreover, in order to achieve significant achievements in the fight against malaria and other mosquito-borne diseases, community participation is an important factor to be taken into consideration [38, 39]. There are a limited number of studies on the assessment of the knowledge and perception of community members about the relationship between livestock keeping and malaria transmission. For example, Nguyen-Tien et al*.* [40] conducted a study to assess the knowledge and practices on the prevention of mosquito-borne diseases (MBDs) in livestock-keeping and non-livestock-keeping communities. The results showed that people in livestock-keeping communities had less knowledge of practices and prevention against MBDs than non-livestock-keeping communities. However, even the study did not assess the knowledge and perception of community members on the relationship between livestock keeping and malaria transmission. Despite assessing the effect of livestock on the prevalence of malaria, Hasyim et al*.* [41] did not capture the attitude and perception of community members about how they know and perceive the problem. Whether livestock have zooprophylactic or zoopotentiation effect, the community must be aware of the situation, and a proper understanding of the situation will encourage better malaria control strategies among the community members. In south-eastern Tanzania, there are a limited number of studies focusing on the impact of livestock on the distribution and densities of malaria vectors. Therefore, this study investigated the impact of livestock management on malaria transmission risks. It further explored the knowledge and perception of community members towards the relationship between livestock keeping and malaria transmission.

## Methods

### Study area

**Fig. 1:**
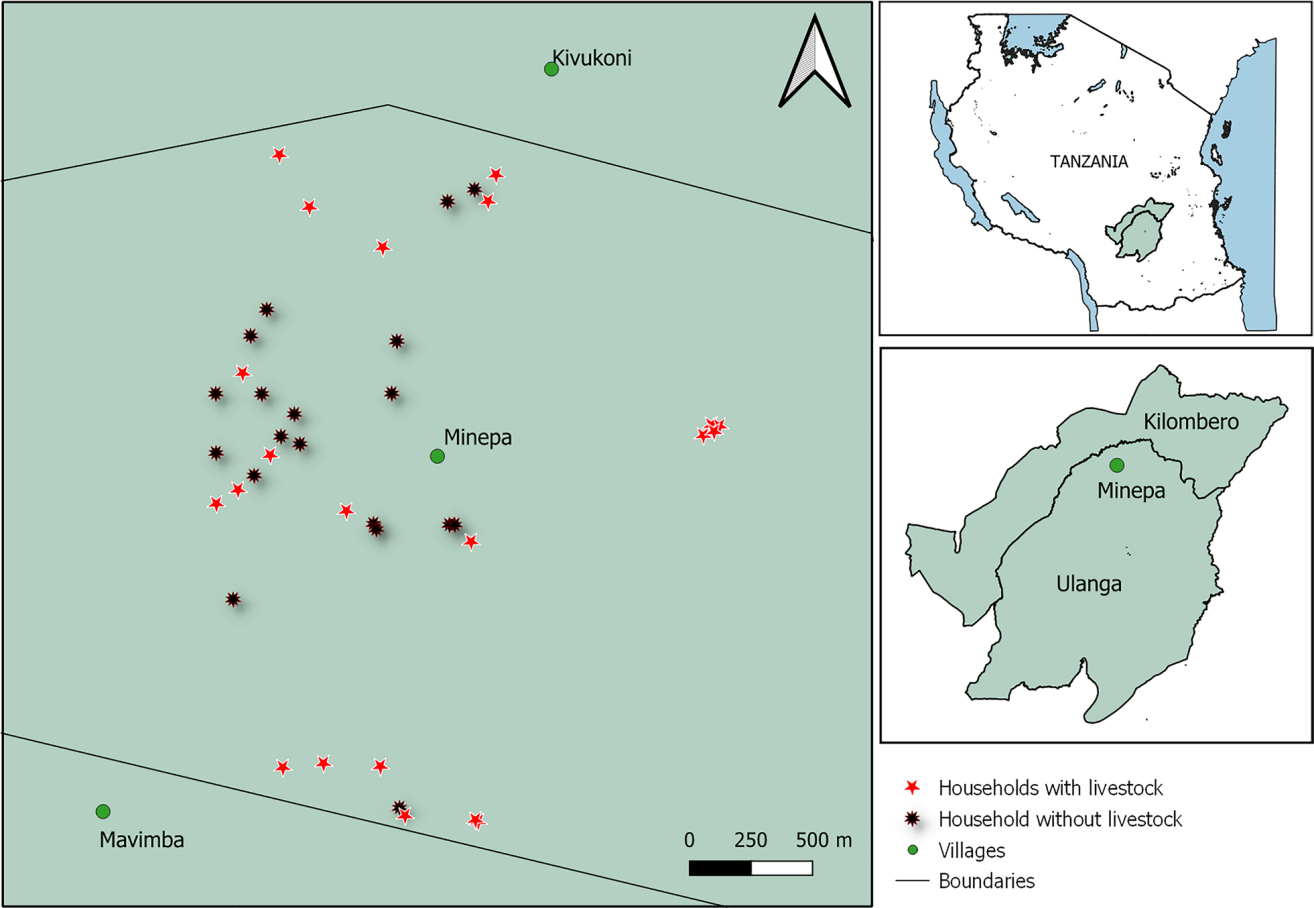
A map showing the household distribution in the study area

This study was conducted in Minepa village (8.21°S to 8.29°S, 36.67°E to 36.71°E), Ulanga district, which is found in Kilombero Valley in south-eastern Tanzania (Fig. 1). The annual rainfall and temperature vary from 1300 to 3600 mm and 15 to 35 ℃, respectively [42]. Most of the residents are small-scale farmers and engage in livestock husbandry, while others engage in small businesses [43, 44]. Common livestock that are kept include cattle, goats, sheep, dogs, pigs, and chickens. The principal malaria vectors are *An. arabiensis* and *An. funestus,* which contribute to more than 80% of contemporary malaria transmission [45–47]. Other *Anopheles* mosquitoes are found in this area, such as *Anopheles coustani, Anopheles pharoensis*, *Anopheles squamosus*, *Anopheles ziemanni,* and *Anopheles wellcomei,* as well as other culicine mosquito species, such as *Mansonia*, *Culex,* and *Aedes* [48]. The main malaria control intervention in the area is the use of long-lasting insecticidal nets (LLINs) [1].

### Selection of households

**Fig. 2:**
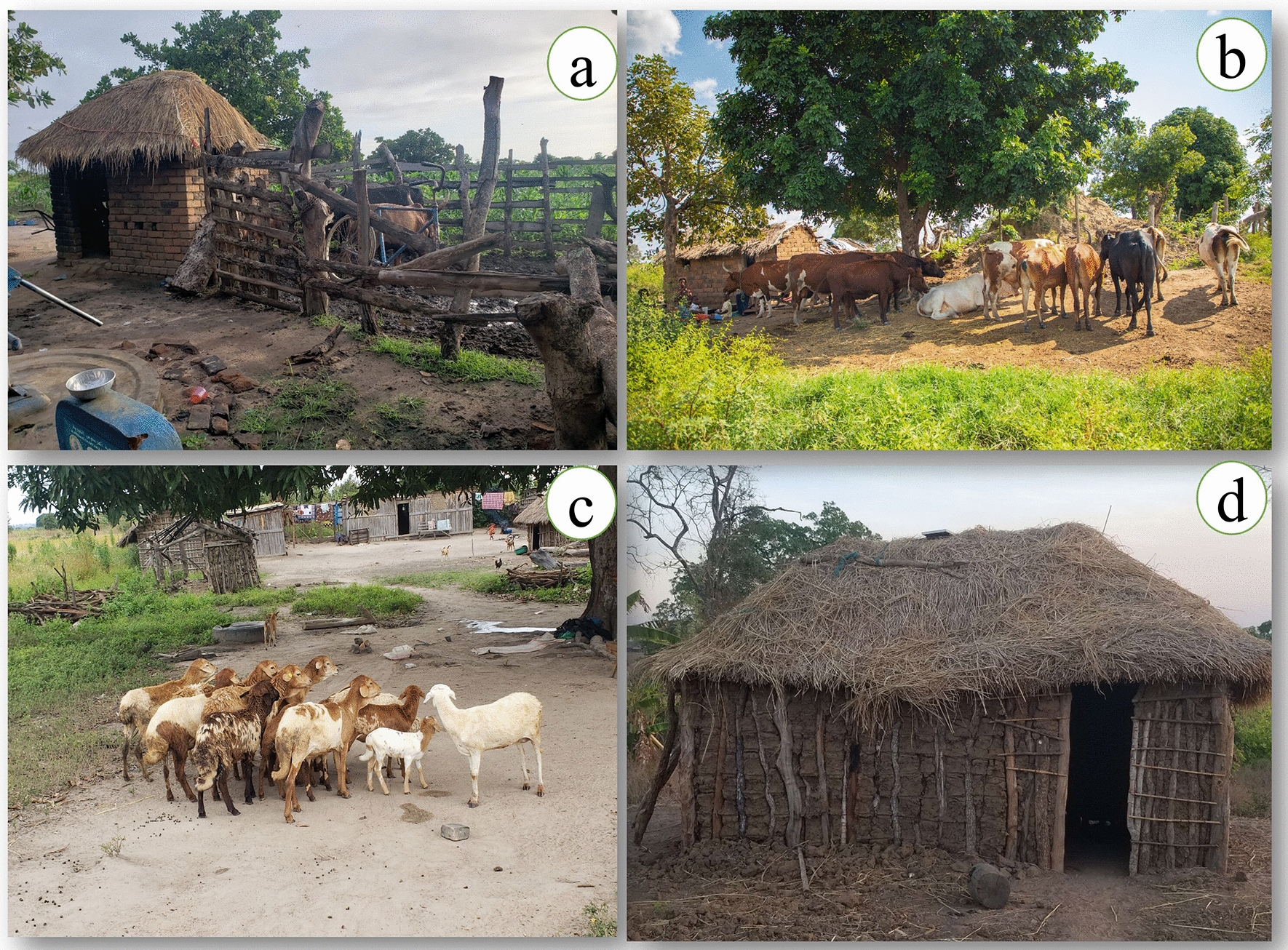
A pictorial representation of the common types of livestock that are kept in the study area village (**b** and **c**) and sample houses that are present in a study area (**a** and **d**)

A total of 40 households were randomly selected; out of which 20 kept livestock (Fig. 2) and the other 20 had no livestock. Selection was conducted in December 2022 for 3 days with the help of village leaders and study volunteers. All selected houses were classified according to their housing characteristics such as door, window, eave space, floor and roof status. Also, details on number of household occupants and distance from household to livestock sheds were observed. All livestock present in selected households were observed and recorded. The common livestock observed were cattle, sheep, goats, dogs, pigs and poultry (chicken and ducks). In some occasions where livestock were kept at different distances, an average distance was calculated and recorded as described by previous study [49]. The chickens and ducks were observed to be kept mostly indoors and sometimes outdoors.

### Mosquito collection

**Fig. 3:**
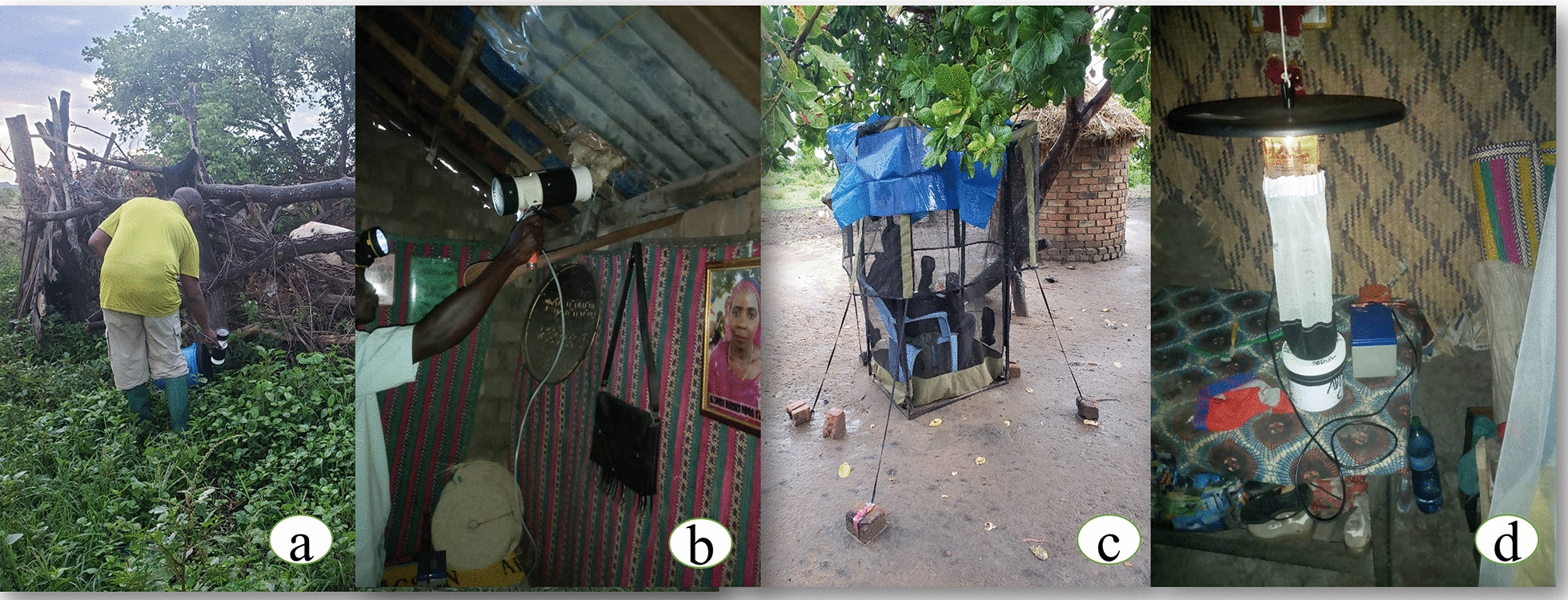
Mosquito traps which were used for mosquito collections; **a** a person collecting resting mosquitoes from resting bucket using prokopack aspirator near cattle shed (**b**) a volunteer collecting resting mosquitoes indoor using a prokopack aspirator (**c**) a double net trap (**d**) a CDC-light trap

Each household was visited once per week from January to April 2023, for a total of 16 nights of mosquito collection per house. Mosquito collection was done indoors and outdoors. Indoor mosquito collection of host-seeking mosquitoes was done using Centers for Disease Control and Prevention (CDC)-Light traps, model 512, John W. Hock Company, Gainesville, FL, USA [50] as shown in Fig. 3d. Resting mosquitoes were collected using a Prokopack aspirator (Fig. 3b) (model 1419, John W. Hock Company, Gainesville, FL, USA) [51, 52]. All selected households were provided with a new, intact, unimpregnated bed net, and CDC-Light traps were set approximately 1.5 m from the ground adjacent to the beds where the protected household occupants slept from 18:00 to 06:00 h. To allow mosquito solicitation, the CDC-Light traps were connected to 12 V batteries. Aspirations were done using Prokopack aspirators, which were connected to 12 V batteries. On some occasions, aspirations were not possible to be conducted indoors because households’ owners were not available to grant permission to do aspirations indoors; they left their houses very early in the morning for agricultural activities. Indoor aspirations were done from 06:00 h to around 08:00. The period of aspiration per household lasted for up to 10 min, depending on the size of the rooms and houses where aspiration was done.

Outdoor collection of hosts seeking mosquitoes was done by well-trained volunteers using human-baited double net traps (Fig. 3c) and traditionally-made mouth aspirators [53]. Outdoor resting mosquitoes were collected using resting buckets. The double net traps were set outside each selected house from 18:00 to 06:00 h by two volunteers, one from 18:00 h to midnight and the other from midnight to 06:00 h. All mosquitoes collected from double-net traps were kept in paper cups covered with a small cloth with small holes. The double net traps were used as an alternative to the human landing catch (HLC) because they protect volunteers from mosquito bites, which may accelerate malaria transmission [54]. Resting buckets (20-L volume) covered with black cloth inside were placed 5 to 10 m away from selected houses from 18:00 h to 06:00 h to allow mosquitoes to rest after night-time activities. In the morning, from 06:00 h to around 08:00 h, resting mosquitoes were collected from resting buckets using a prokopack aspirator. One resting bucket was placed in each selected house during the night of collection, but in houses with livestock, one additional bucket was placed around the livestock sheds (Fig. 3a). The resting buckets were laid on their sides and left open during the night of collection.

### Mosquito identification

All mosquitoes collected were killed using petroleum fumes. Female *Anopheles* mosquitoes collected were morphologically identified by taxa and sex levels using key to the females of Afrotropical *Anopheles* mosquitoes [55] then classified according to their abdominal status as unfed, partly fed, fed, and gravid. *Anopheles* mosquitoes were kept individually or pooled in 1.5 ml Eppendorf containing silica gel desiccant. Every tube was assigned a unique identification number and placed inside storage boxes that included details such as the village name, house number, trap location, species name, and date. These samples were prepared for further laboratory analysis. A sub-sample of *An. gambiae s.l.* and *An. funestus* group were submitted to the Ifakara Health Institute (IHI) laboratory for polymerase chain reaction (PCR) identification of sibling species using a protocol developed by Scott et al*.* [56] and Koekemoer et al*.* [57], respectively.

### Blood meal identification

Fed mosquitoes’ abdomens were removed using forceps and blood antigen (protein) was tested using five different blood sources namely; human, bovine, goat, dog, pig, and chicken using ELISA technique in the laboratory to identify blood meal sources following the procedure described by Chow et al*.* [58].

### Qualitative data collection

#### In-depth interviews

A qualitative assessment was conducted in the village where mosquito collections were conducted. This assessment was done by carrying out in-depth interviews (IDIs) with the household heads where mosquito collections were conducted to assess the knowledge and perception of the community members on the relationship that exists between livestock management practices and malaria transmission. An in-depth interview guide was prepared to capture the following areas of interest: (i) mosquito control and malaria transmission; (ii) livestock keeping practices; (iii) types of pesticides used to protect the animals; (iv) distance between houses and livestock sheds; (v) relationship between malaria transmission and livestock keeping; (vi) impact of livestock keeping on malaria transmission. These IDIs included both households with and without livestock and included male and female participants. Every interview session lasted for 25 to 50 min. All sessions were done in local or village primary school buildings, and on some occasions, the sessions were done within participants’ compounds.

### Statistical analysis

#### Quantitative data analysis

Quantitative data analysis was done using R statistical software version 4.2.1 [59]. Generalized linear mixed models (GLMM) built in template model builder (TMB) following negative binomial distribution were used to model the impact of presence and number of livestock on malaria vector abundance in *glmmTMB* package [60, 61]. In these models, indoor and outdoor mosquito counts were used as response variables whilst presence of different livestock, number of livestock, distance between household and livestock pen, number of household occupants and house characteristics were used as fixed variables. Date of collection and household ID were added as random effect variables to take into account sampling bias and unexplained variation by fixed effect variables. In models involving mosquitoes collected outdoors, the number of mosquitoes collected using resting buckets near livestock pens (NL) were not included. Based on Akaike information criterion (AIC), other variables were not selected for they showed less contribution in the models. All graphs were plotted in *ggplot2* package [62].

Blood indices for different hosts (human, bovine, goat, dog, and chicken) were calculated and compared for mosquitoes collected from houses with and without livestock. The host blood indices were obtained using the following formula:

$$Hos{t}{\prime}s blood index= \frac{Total mosquitoes with specific host{\prime}s blood meal}{Total mosquitoes examined}*100$$

Also, a multinomial logistic regression model was used to assess the influence of livestock and location on mosquitoes’ ability to get human blood. In this model, a blood meal with three levels (human, other hosts, and mixed blood meal) was used as a response variable, while a household’s livestock status and location (indoors or outdoors) were used as predictor variables. This model was implemented using the *multinom()* function from the *nnet* package [63].

#### Qualitative data analysis

Audio data from IDIs were transcribed and then translated from Swahili to English and notes taken during the discussion were also included in the written transcript. Before analysis, the data was checked to assess if it was well translated and documented. The data were analysed using the computer software package for qualitative analysis (Nvivo software version 13) [64] through thematic analysis. The codebook was developed using inductive coding methods. The main themes developed during the analysis were: (i) knowledge about mosquito and diseases they transmit (ii) Knowledge on mosquito biting behaviour (iii) Livestock keeping practices (iv) Types of pesticides used to treat animals (v) Relationship between malaria transmission and livestock keeping and (vi) Impact of livestock keeping on malaria transmission.

## Results

### Summary of all mosquitoes collected

A total of 155,752 female mosquitoes were collected. Out of these, 86,491 (55.5%) were collected indoors and 69,261 (44.5%) were collected outdoors. Among mosquito traps, the CDC-Light trap was the most efficient, collecting 76,344 (49%) mosquitoes, followed by the double net trap, which collected 64,656 (41.6%). Resting buckets near the livestock pen collected the least number of mosquitoes (1,733) among the traps. As indicated in Table 1, 31.3% (48,676) were anophelines, and the remaining were culicine mosquitoes’ species. Among the anopheline mosquitoes, *An. gambiae s.l.* was the most abundant, comprising 43,105 mosquitoes (27.7% of all female mosquitoes collected); others were *An. squamosus* (1972), *An. coustani* (1705)*, An. pharoensis* (1686), and *An. funestus* (209). Near the livestock pens, *An. gambiae s.l.* was the most abundant among the *Anopheles* mosquitoes (95% of all *Anopheles* mosquitoes, n = 981), followed by *An. funestus* (2%, n = 33). There were no *An. squamosus* resting around the animal pens. *Culex* species was the most abundant among culicine mosquitoes, comprising 105,093 mosquitoes (67.5% of all female mosquitoes collected); others were *Mansonia* 1,758 (1.1%), *Aedes* 177 (0.1%), and *Coquilletidia* species 47 (0.03%). This information is presented in Table 1.

**Table 1:** Summary of total number of mosquitoes collected using different traps

Mosquito species	CDC-LT	DN-Trap	Prokopack	Resting bucket-NH	Resting Bucket-NL	Total (%)
*An. gambiae s.l*	22,674	17,143	1079	1228	981	43,105 (27.7)
*An. funestus*	142	85	18	31	33	309 (0.2)
*An. pharoensis*	613	1060	11	1	1	1686 (1.1)
*An. coustani*	1045	637	7	5	11	1,705 (1.1)
*An. squamosus*	1242	625	4	0	1	1,872 (1.2)
Total anophelines	25,716	19,550	1,119	1,265	1,027	48,677 (31.3)
*Culex* spp.	49,871	43,966	9008	1554	694	105,093 (67.5)
*Mansonia* spp.	658	1033	16	40	11	1,758 (1.1)
*Aedes* spp.	91	76	4	5	1	177 (0.1)
*Coquilletidia* spp.	9	31	0	6	1	47 (0.03)
Total culicines	50,629	45,106	9,028	1,605	707	107,075 (68.7)
Overall	76,344	64,656	10,147	2,870	1,734	155,752 (100)

*CDC-LT* Centre for Disease Control and Prevention-Light trap, *DN* Double net, *NH* Near Houses, *NL* Near Livestock pens

### Molecular identification of mosquito species

A total of 4,068 mosquitoes were submitted to the laboratory for mosquito identification of sibling species. Among all *An. gambiae s.l.* examined for sibling species identification, 98% (n = 3991) of these samples were successfully amplified. Among identified mosquitoes, majority of them were *An. arabiensis* (99.97%, n = 3990), except for only one sample that was identified to be *Anopheles quadriannulatus* collected from a house with livestock. A total of 81 *An. funestus* mosquitoes were analysed for sibling species composition, out of which 87% (n = 67) were amplified. Out of these, 64.2% (n = 43) were identified as *Anopheles rivulorum*, 31.3% (n = 21) were *An. funestus s.s*., and 4.5% (n = 3) were *Anopheles leesoni.*

### Common livestock found in the study area

During this study, most of the livestock corralled in the selected households were poultry (43%, n = 267), followed by medium-sized animals (37.2%, n = 231), which include sheep, goats, dogs, and pigs, small animals such as cats (1.3%, n = 8) and large-sized animals, specifically cattle (18.5%, n = 115). This is shown in Table 2. Small animals were not very common.

**Table 2:** Number of livestock present in selected households understudy

Category	Animals’ types	Total (%)
Large size	Cattle	115 (18.5)
Medium size	Sheep	65 (10.5)
	Goats	78 (12.6)
	Dogs	30 (4.8)
	Pigs	58 (9.3)
Small size	Cats	8 (1.3)
Poultry	Chicken	267 (43)
Total		621

### Abundance of host-seeking mosquitoes in houses with and without livestock

**Fig. 4:**
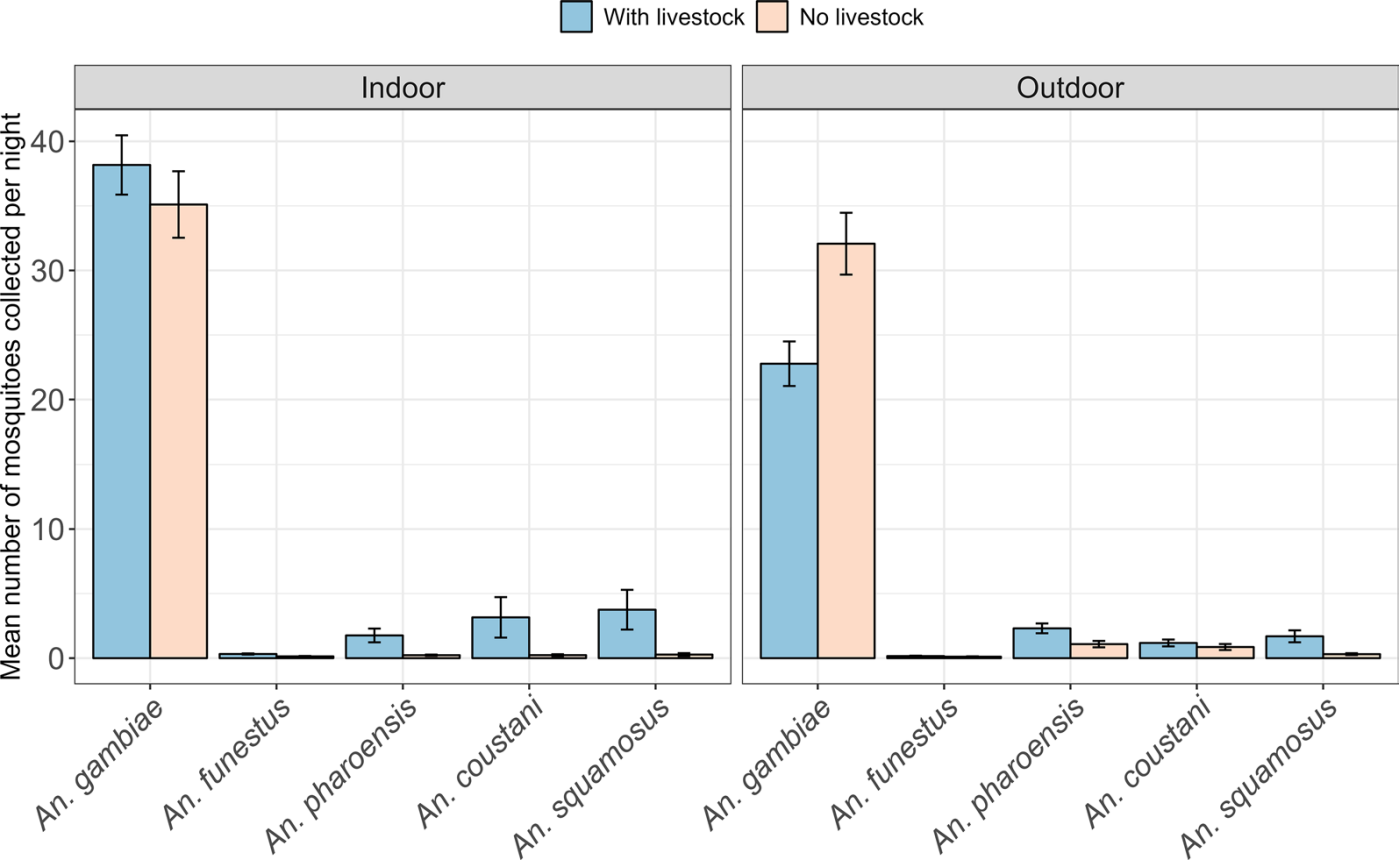
Abundance of host-seeking malaria vectors in houses with and without livestock

The mean number of *An. gambiae s.l.* host-seeking mosquitoes collected by CDC-light traps indoors in houses with livestock was 38.9 ± 2.32 SE, while in houses without livestock, the mean catches were 35.3 ± 2.54 SE (Fig. 4). The mean catches for indoor collection of *An. funestus* host-seeking mosquitoes in houses with livestock were 0.354 ± 0.048 SE, while in houses with no livestock, the mean catch was 0.144 ± 0.029 SE (Fig. 4).

For outdoor collection, the mean number of *An. gambiae* collected in houses with livestock was 24.6 ± 1.73 SE, while in houses without livestock, the mean catch was 31.9 ± 2.33 SE (Fig. 4). The outdoor mean catch of *An. funestus* in houses with livestock was 0.228 ± 0.038 SE, while for houses without livestock, the mean catch was 0.129 ± 0.028 SE (Fig. 4).

**Fig. 5:**
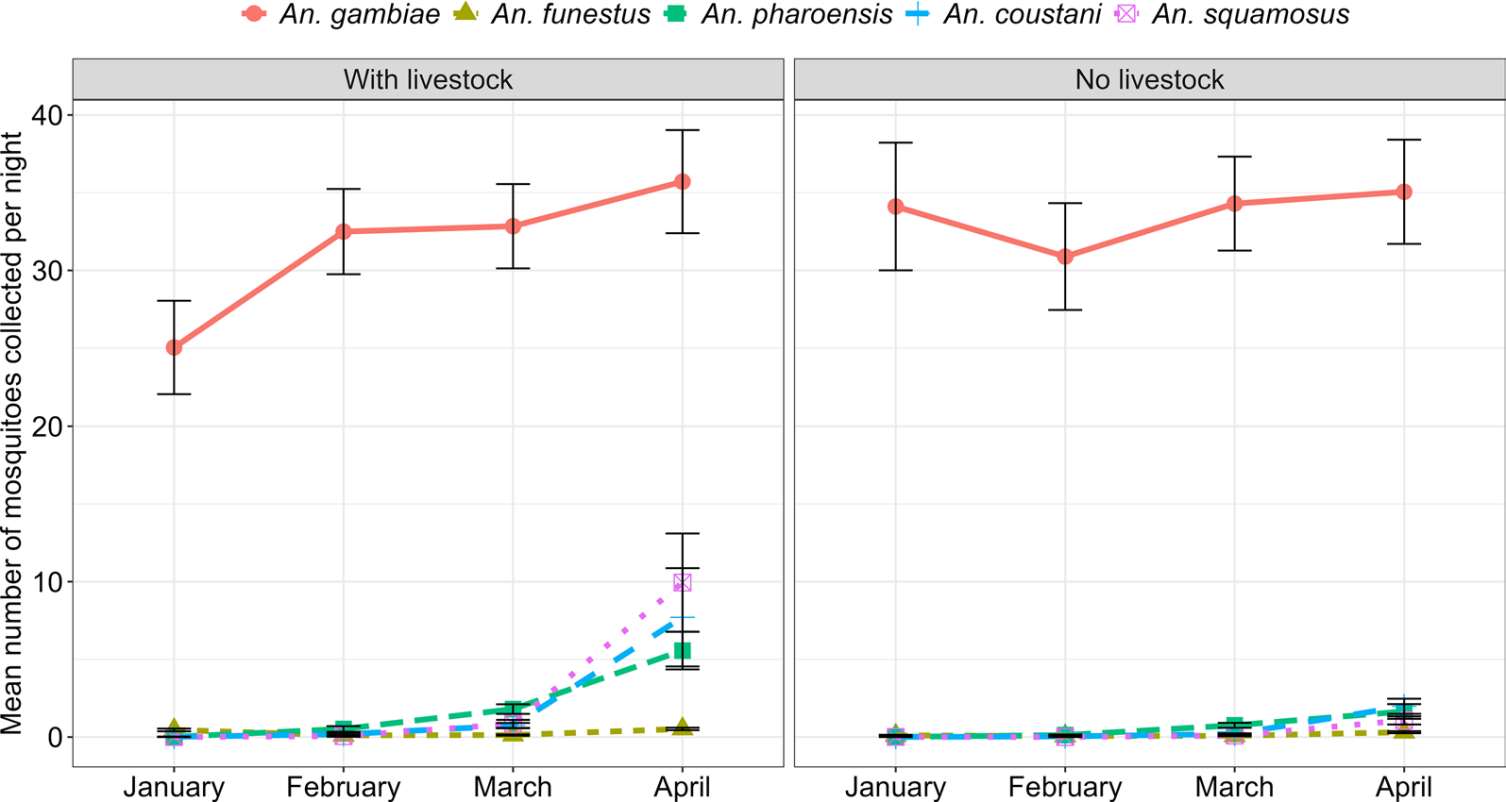
Trend of mosquito abundance in houses with and without livestock in different months during the study period

There was a slight increase in the mean number of *An. gambiae s.l.* in houses with livestock from January to April, but it was somehow constant in houses without livestock. However, for the other *Anopheles* species, an increase in mosquito density was observed in houses with and without livestock between January and April (Fig. 5).

### Abundance of resting *Anopheles* mosquitoes in houses with and without livestock

**Fig. 6:**
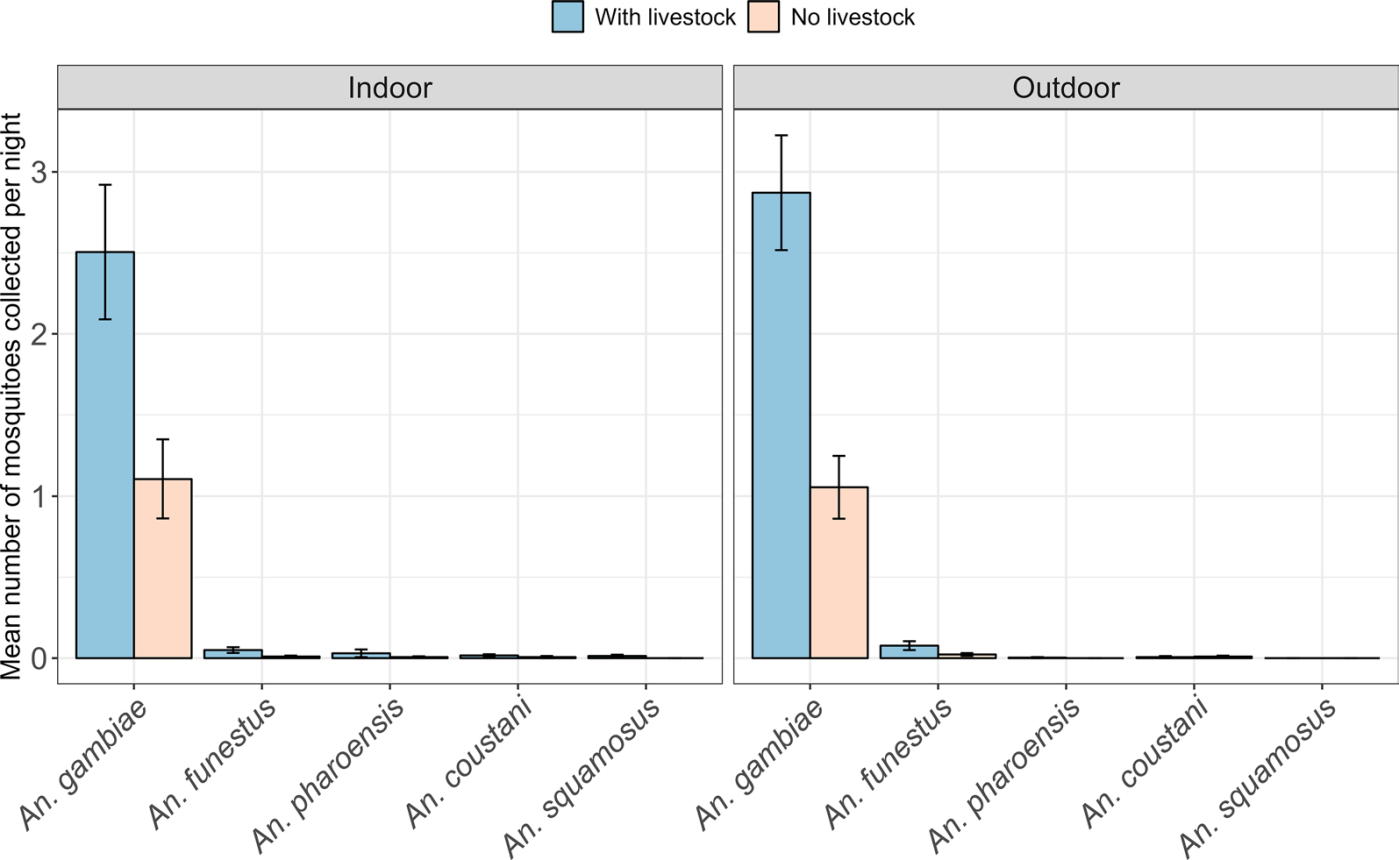
Abundance of resting malaria vectors in houses with and without livestock

More resting mosquitoes were collected indoors and outdoors from houses with livestock compared to houses with no livestock. The mean number of *An. gambiae s.l.* collected indoors from houses with livestock was 2.50 ± 0.416 SE (Fig. 6), while in houses without livestock, the mean number of *An. gambiae s.l.* were 1.11 ± 0.243 SE. For outdoor collections, the mean number of *An. gambiae s.l.* collected from houses with livestock was 2.87 ± 0.355 SE, while in houses without livestock, the mean number of *An. gambiae s.l.* were 1.06 ± 0.194 SE (Fig. 6). The mean numbers of other resting malaria vectors, such as *An. funestus, An. coustani, An. pharoensis,* and *An. squamosus* were marginally less than zero indoors and outdoors. Thus, *An. gambiae s.l.* were the most abundant malaria vectors indoors and outdoors. As it is shown in Fig. 6, the indoor and outdoor collections of *An. gambiae s.l.* and *An. funestus* shows that there were more mosquitoes resting in households with livestock than in houses with no livestock.

### Impact of livestock on the abundance of *Anopheles* gambiae s.l. mosquitoes

The indoor density of *An. gambiae s.l.* increased significantly in households with 11–15 cows (RR = 2.5300, 95% CI 1.225–5.244, *p* = *0.012)*, more than 5 goats (RR = 2.656, 95% CI 1.066–6.619, *p* = *0.001)*, 11–20 chickens (RR = 2.18, 95% CI 1.250–3.803, p = *0.006),* and more than 20 chickens (RR = 1.9214, 95% CI 1.0344–3.5689, *p* = *0.039)* (Table 2). A decrease in *An. gambiae s.l.* catches was revealed to be associated with the presence of 1 to 3 pigs (RR = 0.3444, 95% CI 0.1909–0.9886, *p* = *0.047)* and more than 10 pigs (RR = 0.3344, 95% CI 0.1164–0.9495, *p* = *0.040)* (Table 2).

The outdoor density of *An. gambiae s.l.* was 2 times higher in houses with 11 to 15 cows than in houses with no cows (RR = 2.059, 95% CI 1.056–4.015, *p* = *0.034).* The presence of more than 5 sheep increased the number of *An. gambiae s.l.* approximately 2 times more than households with no sheep (RR = 1.840, 95% CI 1.091–3.100, *p* = *0.022*2.550, Thus, in outdoor collections, only cattle, and sheep were significantly associated with the increase in the number of *An. gambiae s.l.*, but not goats, pigs, or chickens, which had an impact on indoor density (Table 2).

### Impact of livestock on the abundance of *Anopheles funestus s.l.* mosquitoes

The indoor density of *An. funestus s.l.* increased significantly when there were 1 to 5 cows (RR = 3.438, 95% CI 1.3418–8.8098, *p* = *0.010)* and 11 to 15 cows (RR = 2.257, 95% CI 1.623–11.590, *p* = *0.004)* (Table 3). Likewise, the presence of more than 10 chickens, for instance, 11 to 15 (RR = 6.003, 95% CI 2.227–16.180, *p* < 0.001) and more than 20 chickens (RR = 3.055, 95% CI 1.188–7.555, *p* = *0.021),* increased indoor *An. funestus s.l.* densities (Table 3). The number of sheep, goats, and pigs did not have a significant impact on the number of *An. funestus s.l.* collected indoors (*p* > 0.05).

**Table 3:** Statistical significance of the impact of the number of livestock on the number of *An. gambiae s.l.* mosquitoes in houses with and without livestock indoors and outdoors

Livestock type	Number of livestock	Indoor		*p*	Outdoor		*p*
		RR	CI		RR	CI	
Cattle	No cattle	1			1		
	1–5	1.377	0.640–2.962	0.414	0.523	0.274–1.001	1.050
	6–10	1.296	0.756–2.219	0.346	1.283	0.800–2.059	0.301
	11–15	2.535	1.225–5.244	0.012	2.059	1.056–4.015	0.034
Sheep	No sheep	1			1		
	1–5	2.548	0.608–0.687	0.201	3.091	0.888–10.758	0.076
	Above 5	2.508	1.424–4.415	0.001	1.840	1.091–3.100	0.022
Goat	No goat	1			1		
	1–5	1.414	0.659–3.031	0.374	1.149	0.615–2.146	0.663
	Above 5	2.656	1.066–6.619	0.036	1.554	0.646–3.741	0.325
Pig	No pig	1			1		
	1–5	0.434	0.191–0.989	0.047	0.520	0.267–1.014	0.055
	6–10	1.612	0.510–5.090	0.416	0.5314	0.178–1.587	0.257
	Above 10	0.332	0.116–0.950	0.040	0.5518	0.196–1.556	0.261
Chicken	No chicken	1			1		
	1–10	1.018	0.355–2.923	0.974	0.564	0.232–1.373	0.205
	11–20	2.180	1.250–3.803	0.006	0.931	0.561–1.546	0.783
	Above 20	1.921	1.034–3.569	0.039	1.632	0.970–2.747	0.065

*Rate ratio, CI* 95% confidence interval,* p* p-value, *Ref* Reference category

The outdoor collection number of *An. funestus s.l.* increased when there were 11 to 15 cows (RR = 3.279, 95% CI 1.404–7.660, *p* = *0.006)*, more than 5 sheep (RR = 3.001, 95% CI 1.582–5.692, *p* = *0.002)*, and above 20 chickens (RR = 2.541, 95% CI 1.378–4.687, *p* = *0.003).* The number of goats and pigs did not significantly influence the number of *An. funestus s.l.* outdoors (p > 0.05) (Tables 3, 4).

**Table 4:** Statistical significance of the impact of the number of livestock on the number of *An. funestus s.l.* mosquitoes in houses with and without livestock indoors and outdoors

Livestock composition	Number of livestock	RR	Indoor	*p*	RR	Outdoor	*p*
			CI			CI	
Cattle	No cattle	1			1		
	1–5	3.438	1.342–8.810	0.010	1.498	0.651–3.446	0.342
	6–10	1.201	0.526–2.746	0.664	1.420	0.716–2.795	0.310
	11–15	2.257	1.623–11.590	0.004	3.279	1.404–7.660	0.006
Sheep	No sheep	1			1		
	1–5	4.650	0.664–32.555	0.122	2.742	0.705–10.656	0.145
	Above 5	2.490	0.939–5.637	0.081	3.001	1.582–5.692	0.002
Goat	No goat	1			1		
	1–5	1.562	0.487–5.014	0.454	1.401	0.621–3.163	0.417
	Above 5	1.322	0.267–6.532	0.732	1.439	0.429–4.828	0.554
Pig	No pig	1			1		
	1–5	0.876	0.206–3.735	0.858	0.904	0.322–2.542	0.849
	6–10	8.261	1.143–9.691	0.036	1.414	0.320–3.240	0.648
	Above 10	0.604	0.086–4.250	0.612	1.053	0.243–4.558	0.945
Chicken	No chicken	1			1		
	1–10	1.823	0.358–9.272	0.469	0.394	0.079–1.982	0.259
	11–20	6.003	2.227–16.180	< 0.001	1.108	0.487–2.518	0.807
	Above 20	3.055	1.188–7.855	0.021	2.541	1.378–4.687	0.003

*RR* Rate ratio, *CI 95%* confidence interval, *p* p-value

### Distance between houses and livestock pens and mosquito density

The distance between livestock pens and houses did not have an impact on the mosquito density in all species except for *An. coustani,* where the mosquito density at a distance of 30 m was significantly less than the density at a distance of less than 11 m (Table 5). This means that there was a high density of mosquitoes in houses where the distance between houses and livestock pens was less than or equal to 10 compared to 11–20 m (RR = 0.113, 95% CI 0.022–0.588, *p* = *0.010)* (Table 5).

**Table 5:** The effect of distance between livestock pens and houses to the indoor density of *Anopheles* mosquitoes

Species	Distance (meters)	RR	CI	*p*
*An. gambiae s.l*	1–10	1		
	11–20	0.703	0.361–1.372	0.302
	21–30	1.374	0.549–3.442	0.497
	Above 30	0.608	0.137–2.701	0.514
*An. funestus s.l*	1–10	1		
	11–20	0.688	0.332–1.426	0.315
	21–30	1.126	0.440–2.879	0.804
	Above 30	0.361	0.061–2.134	0.261
*An. pharoensis*	1–10	1		
	11–20	0.185	0.030–1.147	0.070
	21–30	0.934	0.095–9.185	0.953
	Above 30	0.178	0.004–8.172	0.376
*An. coustani*	1–10	1		
	11–20	0.113	0.022–0.588	0.010
	21–30	0.208	0.024–1.813	0.155
	Above 30	0.098	0.003–3.151	0.189
*An. squamosus*	1–10	1		
	11–20	0.262	0.038–1.811	0.174
	21–30	0.268	0.019–3.829	0.332
	Above 30	0.553	0.012–24.516	0.760

*RR* Rate Ratio, *CI* 95% Confidence Interval*, p* p-value

### Blood meal sources in livestock-keeping and non-livestock-keeping households

A sub-sample of 2,066 female blood-fed *An. arabiensis* were submitted for ELISA blood meal analysis. The overall identification for blood meal was 76.6% (n = 1,583). In houses with livestock, 747 (71.6%) *An. arabiensis* were positive for bovine blood, 225 (21.6%) for human blood only, 1 for goat, 1 for chicken, 45 for mixed blood meal for human and bovine, 4 for mixed blood meal of human, chicken, and bovine, 1 for chicken blood, 4 for mixed blood meals for human and goat, and 4 for mixed blood meal for human and chicken (Table 6). In houses with no livestock, 363 *An. arabiensis* amplified positively for human blood and 159 for bovine blood. Only 11 mosquitoes had a mixture of human and bovine blood; 2 mosquitoes had a mixed blood meal of human and goat; 2 mosquitoes had chicken blood; and 1 mosquito had a mixture of human, bovine, and chicken blood (Table 6). Generally, only 27% of *An. arabiensis* were detected with human blood in households with livestock while 69.9% of mosquitoes from households with no livestock fed on human blood.

**Table 6:** Blood indices of different hosts from *Anopheles arabiensis* mosquitoes

Hosts Blood	With Livestock (n = 1044)	Without Livestock (n = 539)	Overall (n = 1583)
Bovine	747 (71.6%)	159 (29.5%)	906 (57.2%)
Bovine+Chicken	2 (0.2%)	1 (0.2%)	3 (0.2%)
Chicken	1 (0.1%)	2 (0.4%)	3 (0.2%)
Goat	13 (1.2%)	0 (0%)	13 (0.8%)
Human	225 (21.6%)	363 (67.3%)	588 (37.1%)
Human+Bovine	45 (4.3%)	11 (2.0%)	56 (3.5%)
Human+Bovine+Chicken	4 (0.4%)	1 (0.2%)	5 (0.3%)
Human+Chicken	4 (0.4%)	0 (0%)	4 (0.3%)
Human+Goat	3 (0.3%)	2 (0.4%)	5 (0.3%)

**Fig. 7:**
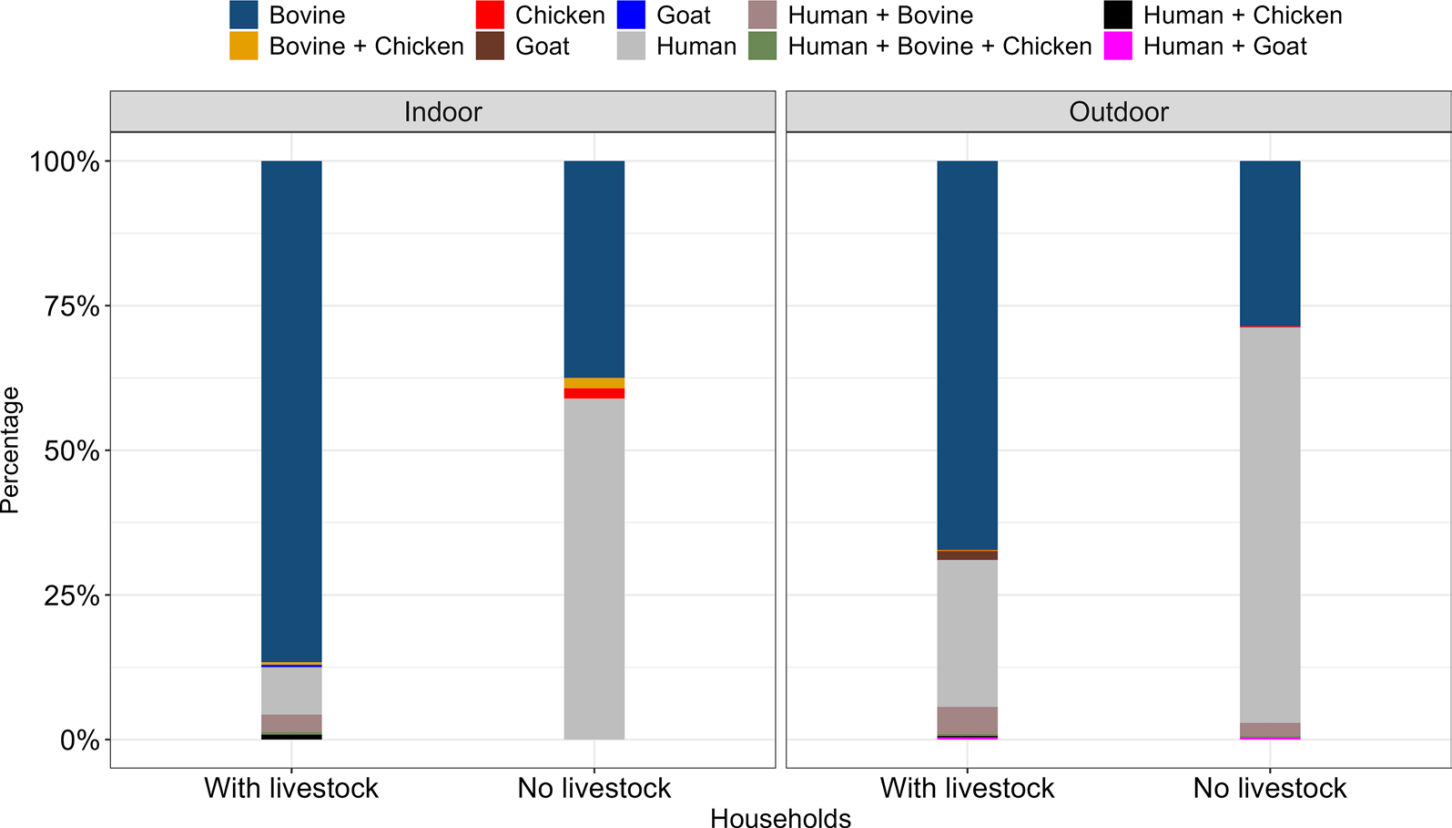
Proportion of blood meal detected from blood fed *An. gambiae* s.l. indoors and outdoors

Indoors, more than 85% of mosquitoes collected indoors in households that have livestock have bovine blood, suggesting that they feed on cows and come indoors to rest (Fig. 7). Also, in households without livestock, more than 60% of mosquitoes have human blood meal, suggesting that the mosquitoes feed on humans only when there are no animals. This could suggest either a lack of alternative hosts prompting mosquitoes to seek human blood or potential preventative measures taken by people to reduce mosquito bites, such as the use of bed nets or repellents. In the outdoors, the majority of the mosquitoes (approximately 75%) in households with livestock had bovine blood meal, suggesting that the livestock attract the mosquitoes outside. In households without livestock, the majority of the mosquitoes had human blood meal, as shown in Fig. 7.

### Impact of livestock on the ability of *Anopheles* gambiae s.l. to acquire human blood

The presence of livestock increased the chance of mosquitoes to feed from other hosts to 7 times more (OR = 7.145, 95% CI 5.618–9.086, *p* < 0.001) than obtaining human blood (Table 7), while mosquitoes were able to obtain a mixed blood of humans from other hosts 6 (OR = 6.350, 95% CI 3.449–11.694, *p* < 0.001) times more than human blood in houses with livestock than houses with no livestock when adjusted to location. This indicates that in households with livestock, there is a high chance of mosquitoes feeding on other hosts other than human blood. Outdoors, mosquitoes were less likely to obtain other hosts’ blood than human blood (OR = 0.369, 95% CI 0.260–0.522, *p* < 0.001) when adjusted to the household’s livestock status (Table 7). This indicates that mosquitoes were able to obtain more human blood meals than other hosts’ blood outdoors compared to indoors.

**Table 7:** Impact of household’s livestock status and location on mosquito ability to feed on human

Variable	Category	Human blood as a reference					
		Other hosts			Mixed (Human+Other)		
		OR	CI	*p*	OR	CI	*p*
Livestock status	No livestock	1			1		
	With livestock	7.145	5.618 – 9.086	< 0.001	6.350	3.449 – 11.694	< 0.001
Location	Indoor	1			1		
	Outdoor	0.369	0.260 – 0.522	< 0.001	0.710	0.339 – 1.490	0.365

*OR* Odds ratios, *CI* 95%Confidence interval, *p* p-value

### Qualitative assessment of knowledge and perceptions of community members about mosquitoes and malaria transmission

#### Demographic description of participants

A total of 20 household representatives participated in the IDIs. The demographic characteristics of the participants are provided in Table 8.

**Table 8:** Demographic information of the study participants

Variable	% (n)
Gender	
Female	60 (12)
Male	40 (8)
Age group	
18–29 Years	40 (8)
30–39 Years	40 (6)
40–49 Years	15 (3)
50 Years and above	15 (3)
Marital status	
Married/Cohabited	60 (12)
Unmarried	35 (7)
Widowed	5 (1)
Educational status	
No formal education	25 (5)
Primary	50 (10)
Secondary and above	25(5)
Main occupation	
Farmer	95 (19)
Business	5 (1)
Household size	
1–3 people	30(6)
4–6 people	35 (7)
Above 6 people	35 (7)

Values are reported as %(n)

#### Knowledge about mosquitoes and diseases they transmit

The majority of the participants in the IDIs understood about mosquitoes (90%) and their habitats (85%) but could not identify them by species level (100%). However, all respondents were able to differentiate them by looking at their physical appearance and colours. The participants knew some of the diseases transmitted by mosquitoes and how those diseases are transmitted from mosquitoes to humans. Malaria was the most mentioned disease by all the participants among all mosquito-borne diseases. The majority of participants sought services in health care centres (85%), but a few participants reported self-medicating (15%). This is shown by the participants below:

“*What I know about mosquitoes is that these are insects that transmit diseases such as malaria and lymphatic filariasis.”* (Female, 34 years)

*“Mosquitoes are found in a variety of habitats; they first lay their eggs in a wet environment, and when they learn to fly, I believe they migrate to populated areas in search of blood.”* (Female, 28 years)

#### Knowledge on mosquito biting behaviour and mosquito control

The majority of the participants reported staying outside before going to sleep (90%) since some of their houses were so small that they only used them for sleeping. Also, all participants responded that they were being bitten by mosquitoes both indoors and outdoors, especially from 7 to 10 p.m. They indicated that they usually sleep around 9:00 p.m. and wake up around 5:00 a.m. They also said there is a large mosquito density outdoors compared to indoors (60%). Almost all participants (95%) reported that malaria incidences keep decreasing every year due to the use of mosquito control interventions, particularly bed nets. They also reported that other vector control tools should be added in line with mosquito bed nets to maximize protection against vector-borne diseases. Examples of these responses are well illustrated by the participants below:

*“Depending on the tasks we have to complete, we occasionally go to bed early and occasionally stay up late. This causes a change in our sleeping patterns from day to day. The majority of the time, if we can get to bed early, we sleep at nine o'clock; however, if we are late, we sleep at twelve.”* (Female, 24 years)

*"We only use bed nets because we don’t have the ability to buy other interventions to protect ourselves from mosquitoes. We sleep very early because our houses are small; if you stay outside, there are many mosquitoes.”* (Female, 23 years)

*“I heard that there are special insecticides to spray on mosquito breeding habitats. I think this will help to crush the mosquito population to a large extent.”* (Male, 27 years)

#### Livestock-keeping practices and distance between houses and livestock enclosures

The majority of participants (75%) reported that livestock such as cows, pigs, and goats were kept outside the houses, while most chickens were kept inside the houses, sometimes sleeping in the same rooms with people. The distances from the houses and livestock enclosure were reported to range between 5 and 30 m. The number of livestock ranges between 3 and 50 per household, depending on the category of animals kept, as illustrated by the following participants:

“In *this village, animals like cows, goats, pigs, and sheep are normally kept outdoors, but chickens are kept indoors because they are stolen by thieves during the night, especially in the rainy season.”* (Male, 27 years)

*“From livestock sheds to houses, it’s like 5 metres; if livestock sheds are very far from houses, it is difficult to hear thieves when they wish to steal our animals, and that is a basic reason why we keep animals near homesteads.”* (Female, 44 years)

#### Types of pesticides used to treat animals

All participants reported to clean places where they keep their livestock often, and they also reported to clean and treat their animals using pesticides. They reported the use of pesticides to protect their animals against animal diseases and insects and ticks and also added that even the rest of the community do the same. They usually treat their animals at least once every two weeks on average; unfortunately, most of them (70%) fail to mention the name of the pesticide they usually use, and few participants (25%) mentioned ‘*paranex’* as their priority among the pesticides. This pesticide contains cypermethrin (synthetic pyrethroid) as an active ingredient, which is used to control ectoparasites that infect cattle, sheep and poultry. Therefore, it might also have an effect on mosquitoes.

They also reported that treating the animals with pesticides reduced mosquito density in a few days (55%). This is illustrated by the participants as follows:

*“Animals like cows are brought to the pasture and led through a mixture of water and insecticides, but we also occasionally spray them with pesticides right here on the farm. We normally spray it with insecticides every week or every two weeks.”* (Female, 44 years)

*“Once the insecticide is sprayed on the day we spray for mosquitoes, the insects truly vanish. For about three days, there won’t be any mosquito activity, and even if you remain outside, you'll be able to see that there are none. I believe another contributing factor is the smell of the pesticides. The strength of the pesticides seems to weaken the mosquitoes on the day of the spraying, but once it wears off, they return in the same manner.”* (Female, 49 years)

#### Relationship between malaria transmission and livestock keeping

This study found that some of the participants understood the relationship between animal keeping and malaria transmission. They also responded that having many livestock increases the population density of mosquitoes and, hence, increases transmission. They mentioned some of the livestock that can contribute to the increase in mosquito density, such as cattle, chickens, and goats.

*“For instance, mosquitoes frequently attack our co-workers who go into the forest to herd cattle. There are typically a lot of mosquitoes where there are herds of cattle.”* (Male, 48 years)

*“Mosquitoes will inevitably grow in number wherever there are lots of animals. Many animal pens, including those for cows, goats, pigs, and chicken coops, will have a lot of mosquitoes, as you'll notice. If you simply sit outside in areas where these animals are present, you risk getting bitten. Additionally, if your home is poorly constructed, mosquitoes will undoubtedly enter in large numbers. They enter inside to bite people.”* (Female, 24 years)

#### Knowledge on the impact of livestock keeping on malaria transmission

Most participants (95%) responded that knowledge of the impact of livestock keeping on malaria transmission is important to their community. Among the participants, 55% of them reported they didn’t get this information anywhere, but they were wondering what would happen with that large mosquito population density. They reported that they think the rest of the community members lack this information, so it is important to consider providing this information to the community. This is well explained by the participants below:

*“The truth is, in areas where there are a large number of animals, the mosquito density becomes very high. I think if these mosquitoes are infected with malaria parasites, the community members will get infected.”* (Female, 49 years)

*“The biggest challenge here in this village is the level of understanding. I believe that many of our fellow villagers sometimes do not realise the way they go about things. I think you should collaborate with the village government to provide better education, especially once you finish your research, so that you can come and share the results with us and we know what to do.”* (Male, 45 years)

*“I believe the community needs more education on unregulated livestock farming; many herders do not follow proper livestock keeping practices, which is why they contribute to the excessive breeding habitats of mosquitoes, especially during the rainy season. Additionally, many of them do not always clean the cattle sheds, resulting in increased mosquito breeding near households. They should be provided with education to bring about a desired change."”* (Male, 31 years)

## Discussion

The study results show that houses with a high number of livestock, especially cattle, sheep, goats, and chickens, experience a higher density of *Anopheles* mosquitoes indoors and outdoors than houses with no livestock. The *Anopheles* mosquitoes encountered were *An. gambiae s.l.* (particularly *An. arabiensis*), *An. funestus s.l*.*, An. pharoensis, An. coustani,* and *An. squamosus*. Subsequently, houses with livestock have an increased risk of malaria transmission. Although the presence of livestock reduced the proportion of *An. arabiensis* mosquitoes with human blood among houses with livestock compared to houses with no livestock, there were some mixed bloods from different hosts, including humans, cattle, goats, and chickens. In houses with livestock, cattle were the most preferred host (71.6%), while in houses with no livestock, humans were the most preferred host (67.3%) by *An. gambiae s.l.*. The effect of distance between houses and livestock shades on the density of mosquitoes collected indoors was not statistically significant except for *An. coustani,* where there was a significant decrease in mosquito collection when livestock were corralled between 11 and 20 m (*p* = 0.010). A higher density of *Anopheles* mosquitoes was observed in houses with mud walls and thatched roofs than in houses with bricks and iron sheets.

The increase in mosquito density in houses with livestock was hypothesized to be due to various possible reasons: (i) Mosquitoes are attracted to odours produced by livestock such as cattle, goats, and others [12, 14], (ii) livestock offers an alternative blood meal source to host-seeking mosquitoes. This is because normally livestock are not protected against mosquitoes like humans, especially during nights when the animals are not sprayed with insecticides and provide an open alternative blood source to host-seeking mosquitoes [12]. This was also revealed by the detection of bovine and other hosts’ blood from *An. arabiensis* mosquitoes. (iii) Cattle urine has been shown to attract primary and secondary malaria vectors in different settings [65–67] as malaria mosquitoes acquire and allocate cattle urine to enhance life cycle traits [67] which might be one of the reasons for the increased mosquito catches in households with livestock, particularly cattle. Due to that, further studies should be conducted to assess other livestock’s urine and other products that might contribute to the increase in mosquito density. The increase in mosquito density in houses with livestock was also revealed in other studies conducted in different countries, such as Pakistan [68] where there was an increase in human biting rate (HBR) in mosquitoes in the presence of cattle and goats. In Kenya, Minakawa et al*.* [33] showed that the ratio of human density to cow density was positively correlated with the relative abundance of *An. gambiae* larvae in the late rainy period. Furthermore, in Ethiopia, two studies revealed that the presence of cattle in proximity to human dwellings increases the HBR of *An. pharoensis* compared to houses with no livestock [69, 70] The results of this study are contrary to studies that have shown that the presence of livestock, such as cattle, was associated with a significant reduction of *An. arabiensis* mosquitoes indoors and outdoors [30, 35, 49]. These studies did not take into account the number and size of livestock corralled. Therefore, it is important to carefully verify these study results in other settings because *Anopheles* mosquitoes seem to behave differently in different geographical areas depending on other covariates, including climatic conditions such as temperature, humidity, and rainfall, and the availability of hosts [71–75].

In this study, the number of resting mosquitoes in houses with livestock was much higher than houses with no livestock both indoors and outdoors. This correlates with the results of host-seeking mosquitoes, where *Anopheles* mosquitoes were higher in households with livestock. The high density of host-seeking mosquitoes was probably a reason for the high number of resting mosquitoes, most of which were either fed or gravid. This shows similar findings to those obtained by Mayagaya et al*.* [35] who observed that the number of outdoor resting *An. gambiae s.l.* was higher in houses with livestock compared to households without, but differed in indoor collections where the number of *An. gambiae* s.l and *An. funestus* was lower in households with livestock indoors compared to households with no livestock [35]. In a study conducted in southern Malawi [49] reported that the number of indoor resting *An. gambiae s.l.* and *An. funestus* mosquitoes in houses with cattle did not differ from houses without cattle.

The presence of livestock at different distances did not have an impact on the densities of malaria vectors except for *An. coustani,* whereby a decrease in mosquito density was observed when livestock were kept between 11 and 20 m from the house. This indicates that the presence of livestock in close proximity to human dwellings reduces *An. coustani* mosquitoes indoors, probably because mosquitoes are more attracted to animals than humans, so the animals pull them indoors, a situation that was clearly described by Iwashita et al. [36]. A study done in Malawi by Mburu et al*.* [49] showed that the presence of cattle at a variety of distances between houses and cattle sheds reduced the density of *An. funestus* mosquitoes when cattle were kept between 1 and 15 m compared to households without cattle.

In the current study, bovine blood was the most preferred blood source among any other hosts, especially for mosquitoes collected in houses with livestock. This confirms that *An. arabiensis,* which was the most abundant species among the *An. gambiae* complex group, is an opportunistic malaria vector that mostly prefers to feed on cattle’s blood [12, 76, 77]. This tells us that the presence of livestock reduces the human blood index, as described by Mayagaya et al*.* in which the proportion of human blood index of *An. arabiensis* and *An. funestus* was approximately 50% lower in houses with livestock than those without Mahande et al*.* [18] also showed that the HBI in *An. arabiensis* was lower in households with cattle than those without cattle. This portrays the zoophilic behaviour of these mosquitoes as it was observed in the current study. In this regard, despite having a higher mosquito density in households with livestock, the HBI is much lower than in households without livestock indoors and outdoors suggesting that animals might be used to control accessibility of human blood meal in areas where *An. arabiensis* is dominant malaria vector [18, 27] and the application of other interventions such as spraying animals with insecticides [21, 78] together with the use of ITNs and IRS would yield best results in malaria control [36, 37]. Also, surprising results showed that even in houses without livestock, there were mosquitoes with bovine blood and some mixed-blood meals of humans and bovine. This might be due to various possible reasons, such as animals not being zero-grazed and just staying outside the homesteads or the possible flight of mosquitoes between households with livestock and those without. It has been previously reported that blood-fed *An. gambiae* mosquitoes can fly up to 10 kms [79]. Only 76% of blood meals were positively identified, according to laboratory tests, so not all blood meals were detected. This might be caused by a variety of reasons, such as mosquitoes feeding on other vertebrates whose antibodies were not present.

The qualitative part of this study shows that most community members observe their home environments clearly regarding the issue of malaria transmission. Some of the participants were able to identify mosquitoes based on their physical appearance and colours. This knowledge can also be used for mosquito surveillance using a citizen science approach [80], where community members may be professionally trained and can be used to continuously monitor species diversity and densities [81]. This will be useful to track trends in dominant mosquito species and be able to detect invasive species that might come into our societies [82, 83]. Mwangungulu et al*.* [84] showed community knowledge and experiences to be used as a crowdsourcing vector surveillance strategy for identifying areas with a high density of mosquitoes instead of conducting large-scale surveillance.

During the qualitative assessment, it was revealed that the majority of community members spend their early evenings or nights outdoors engaging in different activities such as cooking, relaxing, and playing before going to sleep. This exposes them to early-biting mosquitoes. Similar findings were reported by a study conducted to link human behaviours and malaria vector biting risks, where most of the activities done by community members before bedtime exposed them to malaria transmission risks [85]. Therefore, there should be vector control interventions focusing on controlling early biting, such as the use of repellents [86, 87].

The use of pesticides on animals was one of the key aspects that were observed during IDIs, where most of the participants acknowledged using or seeing others use pesticides to treat animals against diseases and mosquito disturbances. This might increase mosquito resistance against those pesticides, some of which contain pyrethroids. Studies should be conducted to assess the susceptibility status of mosquitoes in livestock-keeping households, similar to what was done in rural Tanzania to evaluate the effect of agricultural pesticides on the susceptibility and fitness of major malaria vectors [42]. Furthermore, the cattle treatment records and its potential impact on the outcome should be explored in future studies.

According to community members’ observations of their environments and ecosystems, houses with livestock around their homesteads seemed to have more mosquito abundance than those with no livestock. This information was corroborated by the mosquito sampling activities, which revealed the same scenario for malaria vectors. Thus, this shows that the knowledge and experience of the community members are important baseline information for conducting further studies regarding the relationship between malaria transmission and other underlying variables, as well as before applying vector control tools. Another study showed that livestock-keeping communities have less knowledge and practices on preventing mosquito-mediated diseases than non-livestock-keeping communities [40]. Therefore, community engagement plays an important role in implementing community-based control interventions against various health issues. Providing and mobilizing knowledge about malaria and its risk factors, such as livestock, will assist in reducing malaria transmissions in our settings [88, 89]. This can be done using different approaches, such as the use of drama [90] to convey information to the public.

Despite achieving study results, this study underwent some limitations. First, the study was conducted in the rainy season only and therefore lacks information on the dry season. Thus, it is important for future studies to incorporate seasonality covariates from different consecutive years in order to draw general conclusions. Secondly, the study did not take into account the micro-climatic factors such as temperature and humidity, which also play an important role in the ecology of malaria vectors [74, 75, 91–93]. Thirdly, the sporozoite infection status of the collected female *Anopheles* mosquitoes was not assessed. This lacks confirmation on where exactly malaria transmission is high, despite the presence and absence of livestock. Fourth, the study focused on malaria vectors only, though in the study area, non-malaria vectors coexist with malaria vectors. It is important for other studies to be conducted to assess the impact of livestock on other non-anopheline mosquitoes. Lastly, the study may be limited by the low number of blood-fed mosquitoes collected, potentially affecting the robustness of conclusions drawn regarding the influence of livestock on malaria transmission.

## Conclusion

The presence of livestock, particularly cattle in close proximity to households increases the density of malaria vectors indoors and outdoors. Animals such as cattle, sheep, goats, and chickens have been identified to attract some *Anopheles* mosquitoes. This increases the risk of malaria transmission if no additional malaria control interventions are introduced or implemented. Although number of *Anopheles* mosquitoes increases in the presence of animals, keeping livestock, especially cattle, would reduce mosquito bites from humans and hence zooprophylaxis. This study suggests several actions to be considered: (i) integrated vector management in all sectors; (ii) livestock-based interventions such as spraying animals with insecticides should be applied in livestock-keeping communities where *An. arabiensis* is a dominant malaria vector; (iii) further studies should be conducted to assess mosquitoes’ resting and host-seeking behaviour in livestock-keeping communities across different seasons. (iv) Studies focusing on the effect of various distances between homesteads and animal pens should be further conducted in order to assess the optimal distance where livestock will be kept to reduce mosquito density around the homesteads. Community engagement and the provision of education to the community on malaria control practices are the key aspects to be considered for achieving a significant result against ongoing malaria transmission.

## Data Availability

Relevant datasets for this study are available from the corresponding author upon request.

## Abbreviations

CDC: Centre for Disease Control and Prevention
ELISA: Enzyme-linked immunosorbent assay
GLMM: Generalized linear mixed model
HBI: Human blood index
IRS: Indoor Residual Sprays
LLINs: Long-lasting insecticidal nets
MBDs: Mosquito-borne diseases
PCR: Polymerase chain reaction
WHO: World Health Organization
